# Impact of COVID-19 at the Ocular Level: A Citation Network Study

**DOI:** 10.3390/jcm10071340

**Published:** 2021-03-24

**Authors:** Miguel Ángel Sánchez-Tena, Clara Martinez-Perez, Cesar Villa-Collar, Cristina Alvarez-Peregrina

**Affiliations:** Faculty of Biomedical and Health Sciences, Universidad Europea de Madrid, 28670 Madrid, Spain; masancheztena@gmail.com (M.Á.S.-T.); claramarperez@hotmail.com (C.M.-P.); villacollarc@gmail.com (C.V.-C.)

**Keywords:** COVID-19, ocular disease, SARS-CoV-2, COVID-19 conjunctivitis

## Abstract

Background: The main objective of this study was to use citation networks to analyze the relationship between different publications on the impact of COVID-19 at an ocular level and their authors. Furthermore, the different research areas will be identified, and the most cited publication will be determined. Materials and Methods: The publications were searched within the Web of Science database, using “ocular”, “SARS-CoV-2”, “ophthalmology”, “eyesight”, and “COVID-19” as keywords for the period between January 2020 and January 2021. The Citation Network Explorer and the CiteSpace software were used to analyze the different publications. Results: A total of 389 publications with 890 citations generated on the web were found. It must be highlighted that July was the month with the largest number of publications. The most cited ones were “Characteristics of Ocular Findings of Patients with Coronavirus Disease 2019 (COVID-19) in Hubei Province, China” by Wu et al., which was published in May 2020. Three groups covering the different research areas in this field were found using the clustering functions: ocular manifestations, teleophthalmology, and personal protective equipment. Conclusions: The citation network has shown a comprehensive and objective analysis of the main studies on the impact of COVID-19 in ocular disease.

## 1. Introduction

Coronaviruses are a member of the Coronaviridae family from the Nidovirales order. The name stems from the presence of crown-like spikes on the surface of the virus, thus called coronavirus. These viruses are tiny (65–125 nm of diameter) and contain a single-stranded RNA as genetic material, with a size that varies from 26 to 32 kilobases (kb) of length. There are four subgroups within the coronavirus family: alpha (α), beta (β), gamma (γ), and delta (δ) [1]. Several coronaviruses can infect human beings, such as the endemic human coronaviruses that exist worldwide (HCoV-229E, HCoV-NL63, HCoV-HKU1, and HCoVOC43) that tend to cause a mild respiratory disease, in addition to the Middle-East Respiratory Syndrome (MERS-CoV) and the Severe Acute Respiratory Syndrome (SARS-CoV) that present a higher case fatality rate [2].

COVID-19 is a severe acute respiratory disease caused by the SARS-CoV-2 virus. On 11 March 2020, it was declared as a pandemic by the World Health Organization (WHO) given its high rate of infection, which represents a great threat to global public health. Scientists are still investigating the emergence and origin of SARS-CoV-2. Furthermore, its zoonotic source of transmission in humans has yet to be confirmed. However, the sequence-based analysis has resulted in bats being considered the key reservoir. Recombination of DNA was found to be involved in the spike glycoprotein that combined SARS-CoV (CoVZXC21 or CoVZC45) with the receptor-binding domain (RBD) of another Beta CoV. Hence, this could be the reason for interspecies transmission and rapid infection. Thus, the glycoprotein spikes on the outer surface of coronaviruses are responsible for the binding and entry of the virus into host cells. RBD is loosely bound between viruses, therefore the virus can infect multiple hosts. Other coronaviruses primarily recognize aminopeptidases or carbohydrates as a key receptor for entry into human cells, while SARS-CoV and MERS-CoV recognize exopeptidases [3,4].

Some recent studies have shown that this new coronavirus strain can lead to conjunctival findings and can be detected in tears and conjunctival secretions [5,6].

SARS-CoV-2 RNA has been detected through a RT-PCR on conjunctival samples in 0% to 15% of infected patients [7,8]. However, and although some studies have described the positivity of the virus genome in tear samples from patients with SARS-CoV-2, the relevance of these findings remains controversial [9]. A total of 40% of patients with a positive conjunctival swab present with symptomatic conjunctivitis. Likewise, the appearance of ocular symptoms is frequently the first symptom, or they can appear along with other systemic pathologies [10]. The notified manifestations and symptoms are as follows: bilateral/unilateral diffuse hyperemia, viscose white secretion in the conjunctival sac, foreign body sensation in the eye, and excessive tearing [11,12].

It must be highlighted that secondary conjunctivitis is the main ocular manifestation in COVID-19 patients and the main viral conjunctivitis is less frequent [13]. The prevalence rate ranges from 0.8% to 31.6% of patients [6,14]. In a retrospective study carried out with 1099 samples from positive COVID-19 patients diagnosed by laboratories of 552 hospitals in 30 Chinese provinces, the conjunctivitis rate was 0.8% [6]. In another study, it was found that 12 out of 38 COVID-19 patients showed conjunctival hyperemia, chemosis, epiphora, or increased conjunctival secretions. Thus, these manifestations were linked to conjunctivitis. However, only two patients yielded a positive RT-PCR finding in their conjunctival swabs [14]. At a later stage, it was proved that a higher prevalence of conjunctivitis can be related to incomplete closure of the eyelids in sedated and semi-conscious patients in the intensive care unit [15].

On the other hand, various studies have shown that epithelial cells from the ocular surface can selectively respond to specific components of ocular pathogenic bacteria by producing pro-inflammatory cytokines. On the contrary, it was shown that they do not respond to non-pathogenic bacteria, which favor colonization by a real microbiota. However, analysis of the composition of the ocular microbiome is essential to understand the pathophysiology of various ophthalmic diseases. Therefore, the alteration of the normal microbiota of the eye may have an important activity as a cofactor in the pathogenesis of ophthalmic diseases. In turn, recently the alteration of the microbiota of other body sites has been considered that it can favor the development of ophthalmic pathologies. In this sense, changes in the composition of the oral and intestinal microbiota have been associated with glaucoma, uveitis, and AMD, respectively. From this method, the analysis of the ocular microbiota is important to improve the knowledge that homeostatic microorganisms have in the prevention of various ophthalmic diseases and thus develop new therapeutic strategies, based above all on the intake of probiotics, to treat ocular pathologies [16].

As a response to this pandemic, and to reduce the referral of patients to hospitals or health care centers, several studies have analyzed the feasibility and effectiveness of telemedicine in ophthalmology (teleophthalmology). Teleophthalmology has been successfully used in hospitalized patients and outpatient centers, and it has been implemented in a variety of socio-economic contexts. Although most of the successful initiatives so far have been focused on the screening and early detection of ophthalmological diseases, teleophthalmology has also a great potential for patients’ treatment and follow-up [17,18].

Citation network analysis is used to search scientific literature on a specific subject. That is, through a single publication, it is possible to find additional and relevant publications to prove qualitatively and quantitatively the relation between articles and authors while creating groups [19]. Furthermore, it allows to measure the most cited publication within each group, as well as to study the development of a research field or to focus the search on a specific subject [20,21].

Thus, given the increasing number of publications about COVID-19 and its ocular consequences, this study aims is to identify the different research fields and to determine the most cited publication. In addition, the relation between the different publications and research groups will also be analyzed through the CitNetExplorer software, whose main objective is to study the development of scientific literature in a research field.

## 2. Materials and Methods

### 2.1. Database

The search of different publications was carried out through the Web of Science (WOS) database, using the following keywords: “ocular”, “SARS-CoV-2”, “ophthalmology”, “eyesight”, and “COVID-19”. These keywords were selected according to the main objective of this study and the fact that they are the most common words in all the different research fields.

As the obtained results had some articles in common, the Boolean operators NOT and AND, and the “*” character were used to find the singular and plural forms of the words. In this way, the words used in the first search were (“ocular” AND “COVID-19”), in the second search (“ocular” AND “SARS-CoV-2” NOT “COVID-19”), in the third one (“Ophthalmol*” AND “COVID-19” NOT “ocular”), in the fourth one (“Ophthalmol*” AND “SARS-CoV-2” NOT “COVID-19” NOT “ocular”), in the fifth search (“Ophthalmol*” AND “The Coronavirus Disease 2019” NOT “SARS-CoV-2” NOT “COVID-19” NOT “Ocular”), and in the sixth (“eyesight” AND “COVID-19” NOT “ocular” NOT “Ophthalmol*”).

Likewise, the search field was selected by Topic, limiting the search by summary, title, and keywords. The selected time interval covered the period from February 2020 to January 2021.

In turn, the Web of Science also makes it possible to add references to the library while conducting bibliographic searches directly in external databases or library catalogs.

Regarding the citation index, Social Sciences Citation Index, Science Citation Index Expanded, and Emerging Sources Citation Index were the employed tools.

On the other hand, due to the different citation methods used by the authors and organizations, the CiteSpace software was used to standardize the data. The search and download date was 23 January 2021.

### 2.2. Data Analysis

The publications were analyzed using the Citation Network Explorer software. This software allows for the analysis and visualization of the citation networks of scientific publications, and it also allows for citation networks to be downloaded directly from the Web of Science. It is also possible to manage the different citation networks including thousands of inter-related publications and citations. This way, researchers can start with a citation network consisting of thousands of publications and, then, focus only on the relevant ones to generate a small subnet of 100 publications about the same topic.

Using Citation score metrics, a quantitative analysis on the most cited publications within a time interval was carried out. This way, both internal connections within the Web of Science database and external connections, that is, considering other databases, were quantified [21].

CitNetExplorer offers several techniques to analyze the different citation networks. The clustering function is achieved by using the formula developed by Van Eck in 2021 [20].

(1) $$V\left(c_{1\;},\ldots ,c_{n}\right)=\sum_{i<j}\delta \;\left(c_{i},c_{j}\right)\left(s_{ij}-\gamma \right).$$

Next, the clustering function was used to assign a group to each publication. Thus, the most interrelated publications tend to be within the same group according to the citation networks [21].

Finally, the main publications were analyzed using the identifying core publications function. This function is based on identifying the publications that are considered as the core of a citation network, that is, the ones that have a minimum of connections with other main publications, to eliminate those that are not important. The number of connections is established by the researchers, so the higher the value of this parameter, the lower the number of core publications [21]. Thus, in this study, we have considered the publications with 4 or more quotes within the citation network.

On the one hand, the drilling down function was used since it allows us to deepen and analyze each group into different levels.

Then again, the CiteSpace (5.6.R2) software was used to conduct the scientometric analysis. This software was developed by Chen Chaomei, it is based on Java, and it is mainly composed of five basic and theoretical aspects: Kuhn’s paradigm in scientific development, the scientific borders theory by Price, ideas organization, the scientific communication theory on the best search of information, and the theory of discrete and reorganized insight units [22,23]. In the scientometric analysis process, there are also some parameter indicators to carry out a specific assessment. The H-Index is a mixed quantitative index suggested by George Hirsch from the University of California, United States. It is employed to assess the number of academic publications and the level of these publications carried out by researchers and organizations. The H-Index indicates that h out of N published articles in a journal have been cited at least h times [24]. The Degree indicates the number of connections among the authors (organizations, countries) in the co-occurrence knowledge graph. A higher value in this Degree indicates more communication and collaboration among the authors (organizations, countries). Moreover, the centrality value measures the importance of the nodes within the collaboration network in the studies, and the average life is a parameter that represents the continuity of institutional studies from a time perspective [22].

## 3. Results

The first articles on the influence of COVID-19 at the ocular level were published at the beginning of 2020, so the period selected for the search was from January 2020 to January 2021. After searching in WoS, 389 publications were found according to title, abstract and keywords, and 890 citation networks.

**Figure 1:**
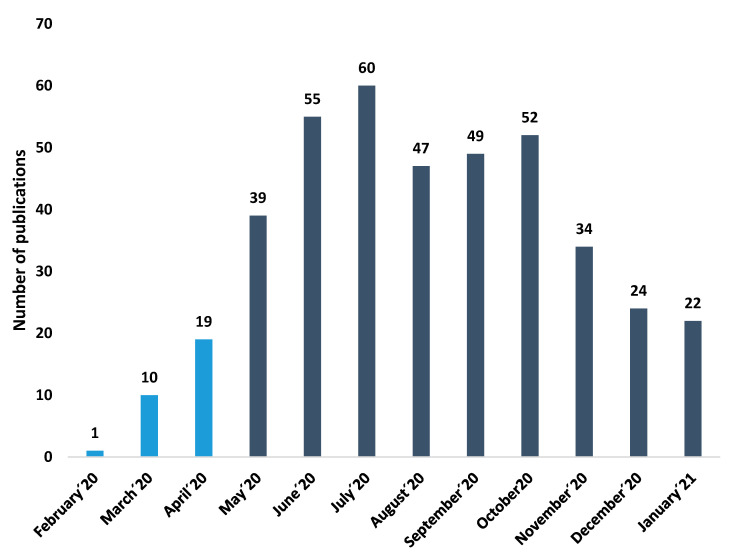
Number of publications per year.

As shown in Figure 1, the number of publications on eyesight and COVID-19 has increased exponentially since May 2020 (February 2020–April 2020: 7.05%; May 2020–January 2021: 92.95%). July was the month with the largest number of publications (60 publications) and 14 citation networks.

### 3.1. Description of the Publications

Of all publications, 53.5% of them were articles, 20.7% were reviews, 12.7% were letters to the editor, 11.8% were “editorial material”, and the remaining 2% were corrections and abstracts of congresses and conferences.

#### 3.1.1. Language and Countries

As for the language of the publications, 93.6% were in English, 2.8% in German, and 1.95% in French. This is because English is one of the most widely used languages worldwide, so researchers who write in English have more opportunities to get published [25]. Simultaneously, it is related to the fact that the growing trend in the number of publications in countries such as the United States or Great Britain has been associated with a combination of factors such as being English-speaking countries and the possible influences between the multiple research groups in the scientific community [26,27].

**Figure 2:**
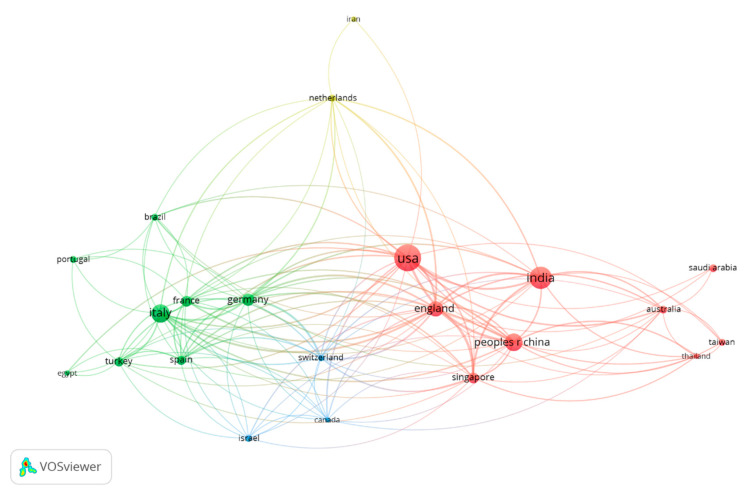
Collaboration between countries.

Therefore, Table 1 and Figure 2 show the countries of publication of the journal with the highest number of citations and the connections in the citations between countries. As shown in Figure 2, the countries with the largest number of publications are the United States (26.1%), India (17.5%), and Italy (13.6%). Figure 2 shows the publications with the highest weight (highest number of citations), as well as the group to which they belong. The color of an article represents the group to which it belongs, and the lines between elements represent bonds.

Table 1 shows the main characteristics of the four most important groups in Figure 2.

#### 3.1.2. Research Areas

The area of research is multidisciplinary; however, the fields of ophthalmology (67.6%) as well as both internal and general medicine (35.4%) (Table 2) are particularly worth mentioning.

#### 3.1.3. Authors and Institutions

As shown in Table 3, the first authors with the highest number of publications in the area of eyesight and COVID-19 were Sharma N (2.3%), Bandello F (2.0%), and Agrawal R (1.8%).

Among the institutions with the largest number of publications (Table 4) are All India Institute of Medical Sciences (3.8%), LV Prasad Eye Institute (3.3%), and Ctr Sight (3.1%).

#### 3.1.4. Journals

Table 5 shows the main journals that have published on eyesight and COVID-19 and the number of publications according to the WoS database. The quartile stated by the Scimago Journal Rank (SJR) was included in the table to introduce the importance and relevance of the top journals that have published the most articles. This ranking was chosen because it was the most widely used in the scientific field. Quartiles are based on the ranking of each journal according to its subject, using as a measure the distribution of the journal’s impact factor for that subject category. The Scimago Journal Rank is a portal of scientometric and informetric indicators that allows researchers to track the performance and impact of their contributions on an international scale, meaning that it measures the scientific influence of journals according to the number of citations. The weighting of citations depends on the subject field and the standing of the citation series [28].

#### 3.1.5. Keywords

**Figure 3:**
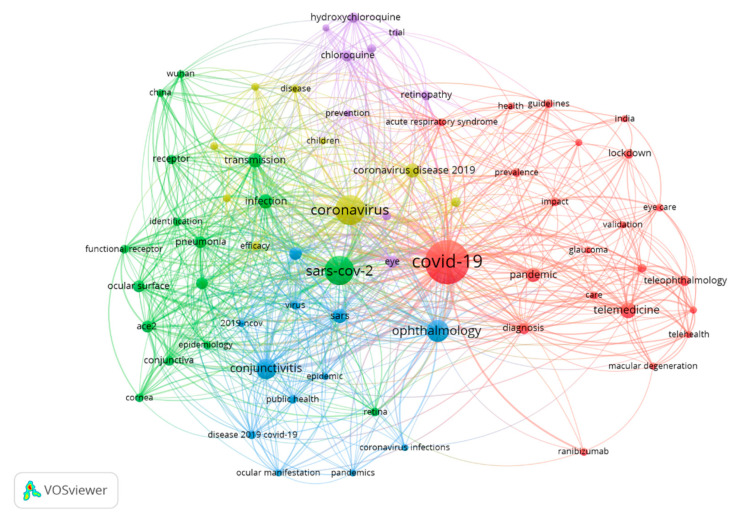
Link between keywords.

On the other hand, the most used keywords were “COVID-19” (175 publications), “Coronavirus” (84 publications), and “SARS-COV-2” (78 publications). Table 6 and Figure 3 show the most used keywords in the most relevant publications and their frequency of appearance in publications with other keywords.

Table 7 shows the main characteristics of the five most important groups in Figure 3.

### 3.2. Most Cited Publications

The most cited article was Wu et al. [14], published in March 2020 with a citation index of 229. This study is based on several cases, which aimed to investigate the ocular manifestations and conjunctival viral prevalence in 38 patients with COVID-19. During the treatment period, ocular signs and symptoms were recorded and analyzed, as well as the results of blood tests and reverse transcriptase-polymerase chain reaction (RT-PCR) of nasopharyngeal and conjunctival swabs for SARS-CoV-2. Among them, 28 patients had positive findings for COVID-19 on RT-PCR of nasopharyngeal swabs, of which two patients were positive for SARS-CoV-2 in their conjunctival and nasopharyngeal samples. In addition, 12 of 38 patients presented ocular symptoms related to conjunctivitis, such as conjunctivitis, including conjunctival hyperemia, chemosis, epiphora, or increased secretions. Patients with eye symptoms were at greater risk of having higher white blood cell and neutrophil counts and higher levels of procalcitonin, C-reactive protein, and lactate dehydrogenase than patients without eye symptoms. Furthermore, 11 of the 12 patients with ocular anomalies were positive for SARS-CoV-2 on RT-PCR of nasopharyngeal swabs.

When analyzing the 20 most cited articles, 18 of them discuss ocular symptoms and manifestations in patients with COVID-19. In turn, two of them attempt to identify a general agreement and provide recommendations for the use of PPE in the most common ophthalmological scenarios (Table 8).

### 3.3. Clustering

The clustering function was used to assign a group to each publication in such a way that the publications which are close within the citation network must belong to the same group. Therefore, each group consists of publications with strong links connecting them in terms of citation connections. In this way, it may be interpreted that a group represents one topic in the scientific literature. To distinguish between groups, different colors were assigned to each of them and the connections among groups are shown using colored lines.

**Figure 4:**
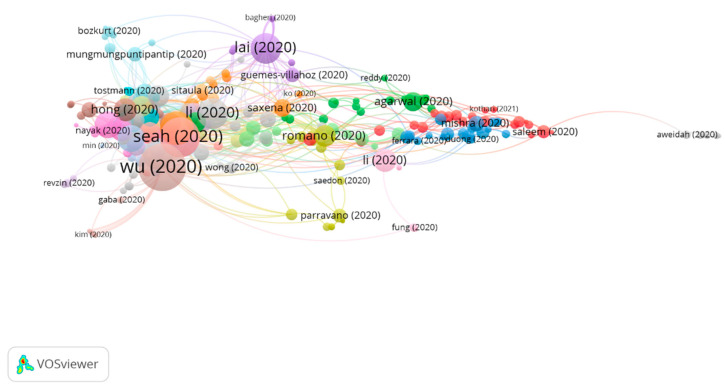
COVID-19 citation networks and vision.

Three groups were found in this analysis, all of them featuring a significant number of publications (Figure 4).

Table 9 shows the information on citation networks for the three main groups, ordered by size, from the largest to the smallest (the remaining articles do not belong to any group. For this reason, they have not been included).

#### 3.3.1. Cluster Group 1

A total of 158 publications and 604 citations in the whole network were found in group 1. The most cited publication was that of Wu et al. [14] published in March 2020 in *JAMA Ophthalmology*, and this also takes the first position in the 20 most cited publications. The articles in this group study the occurrence of manifestations on the ocular surface of patients diagnosed with the Coronavirus 2019 disease. SARS-CoV-2 may cause ocular complications such as viral conjunctivitis in the disease intermediate stage. However, conjunctival swabs may not be useful in the early diagnosis because the virus might not be initially present in the conjunctiva. Furthermore, ocular symptoms have been found to have a relatively low prevalence among COVID-19 patients. Oddly enough, it seems that ophthalmic manifestations are associated with the severity of the COVID-19 disease.

**Figure 5:**
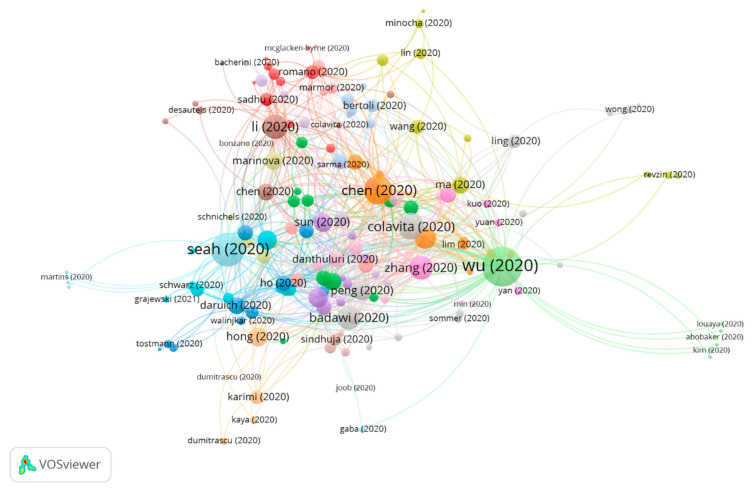
Citation network in group 1.

Likewise, an analysis is also carried out to find out whether the cells on the ocular surface have the key factors required for cell susceptibility to SARS-CoV-2 entry/infection (Figure 5).

#### 3.3.2. Cluster Group 2

A total of 68 publications and 84 citations in the whole network were found in group 2. The most cited publication was that of Saleem et al. [44] published in August 2020 in the *American Journal of Ophthalmology*. That study aimed to revise of the effects of SARS-CoV-2 on the outpatient ophthalmic practice, the value proposal of telemedicine, the implementation methodologies for teleophthalmology, and the accelerated future of telemedicine. Telemedicine and teleophthalmology have existed for several years, yet these have gained increasing importance in the current scenario of the COVID-19 pandemic. Even though the technology for remote ophthalmic follow-up is not available yet, it seems that patients are willing to embrace this approach. This crisis is likely to promote innovation which will transform the delivery of personal care services. In regards to ophthalmology, this might mean more precise home tests. For example, smartphone cameras’ focus is likely to be improved.

Remotely operated slit lamp devices, non-mydriatic fundus cameras, and optical coherence tomography devices could become increasingly available in public areas. Ophthalmologists could schedule follow-up telephone calls and video calls with patients to discuss test results, verify treatment adherence, or categorize patients.

It is important to acknowledge that certain subspecialties are more susceptible to telemedicine consultations than others. Oculoplastics, neuro-ophthalmology, and pediatrics seem more appropriate for video calling as most of their tests can be conducted externally. Anterior segment pathology is more difficult to approach from the perspective of telemedicine unless the pathology is visible upon external examination. In turn, video calling could prove particularly useful in the management of glaucoma for medication reconciliation and the assessment of glaucoma drug tolerance. In regards to the retina, the sending of images has already been established in certain contexts.

**Figure 6:**
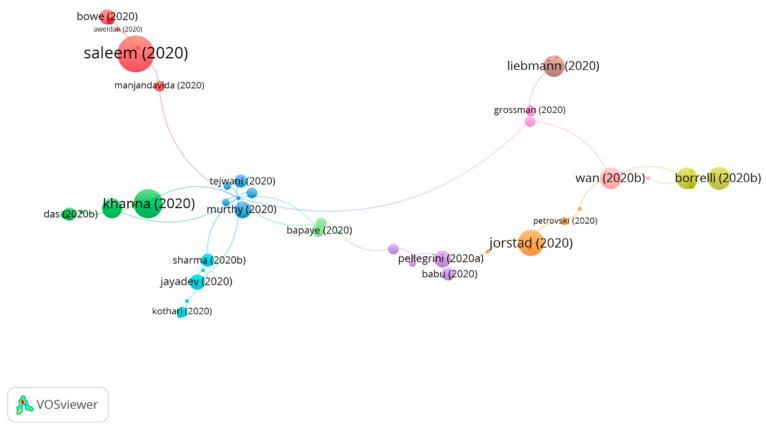
Citation network in group 2.

Therefore, the papers in this group analyzed how teleconsultations seem to be here to stay, even beyond the pandemic, as well as addressing how the clinical practice will need to evolve according to the standards of telemedicine (Figure 6).

#### 3.3.3. Cluster Group 3

In group 3, 60 publications and 87 citations were found in the whole network. The most cited publication was that of Lai et al. [30] published in May 2020 in *Graefe’s Archives for Clinical and Experimental Ophthalmology*. This publication aimed to share a local experience to intensify control measures for infections in ophthalmology and thus minimize COVID-19 infection among both health workers and patients. To achieve this, a hierarchy of three levels was established for control measures. First, as a method for administrative control and to reduce patient attendance, text messages were sent containing the phone number for inquiries that the patients should contact to reschedule appointments or request medication refills. To minimize COVID-19 cross-infection, a classification system was implemented to identify patients with a fever, respiratory symptoms, acute conjunctivitis, or recent trips to areas with outbreaks, and these patients were encouraged to postpone their appointments for at least 14 days. The micro-aerosol generating procedures were avoided, such as contactless tonometry or surgery under general anesthesia. Nasal endoscopies were also avoided, as these were likely to provoke sneezing, therefore generating droplets. Likewise, all elective clinical services were suspended, and all clinical personnel received training as to how to control infection. Secondly, in regards to environmental control, and to reduce COVID-19 droplets transmission, measures were implemented such as installing protective screens in slit lamps, disinfecting all equipment frequently, and providing ocular protective equipment to all staff, who were also advised to check their body temperature before going to work and report any symptom of infection in their upper respiratory tract, vomiting, or diarrhea. Thirdly, universal mask-wearing was promoted as well as hand hygiene and the appropriate use of personal protective equipment (PPE).

**Figure 7:**
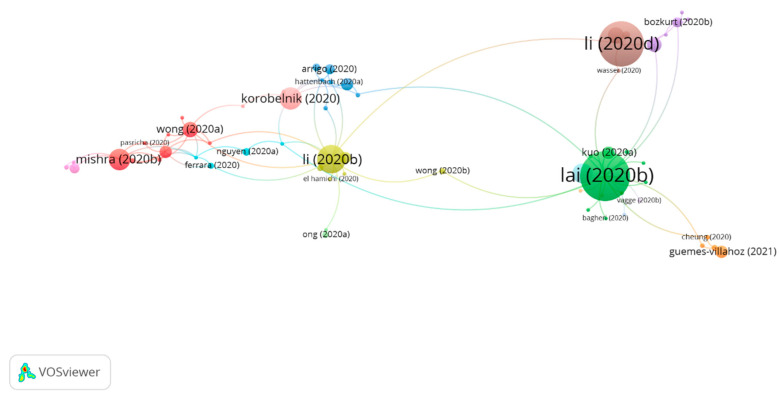
Citation network in group 3.

Therefore, publications in this group attempt to identify consensus and provide advice regarding PPE for the most common ophthalmic scenarios. This is a result of the fact that the shift in clinical practice worldwide appears to suggest that there is a need for a network of international ophthalmic partners to, based on evidence, reach a consensus on the protocols for risk mitigation that adequately protect patients, staff, ophthalmologists, and the general public. Consensus with regards to operational matters, such as cleaning protocols for instruments and the adequate use of PPE in different circumstances, will help systems to decide on the appropriate assignment of very scarce resources (Figure 7).

When analyzing the relation between the most cited publications of each group, no connection was found among the groups. That means that each group deals with topics that are different from those of others.

#### 3.3.4. Subclusters Group 1

**Figure 8:**
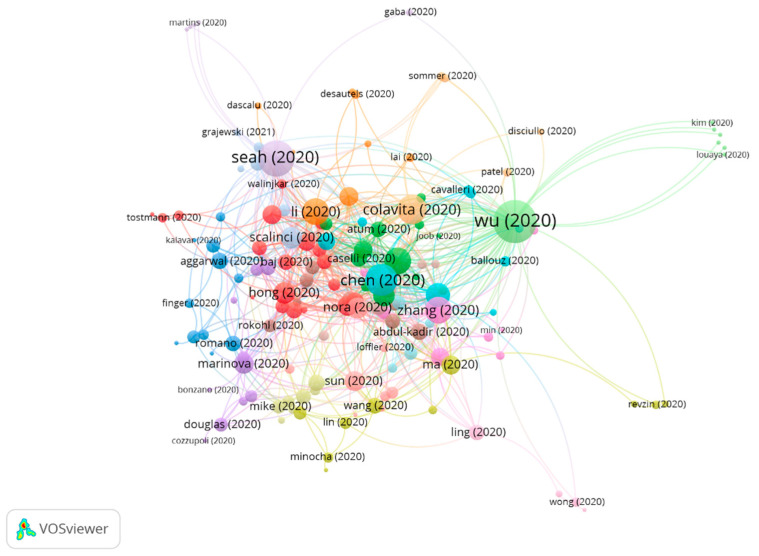
Citation network of group 1 subclusters.

Due to the high number of articles in group 1 and the various research topics, the subclusters of group 1 have been analyzed. Three subclusters were found (Figure 8), all of them with a significant number of publications (Table 10).

### 3.4. Core Function

**Figure 9:**
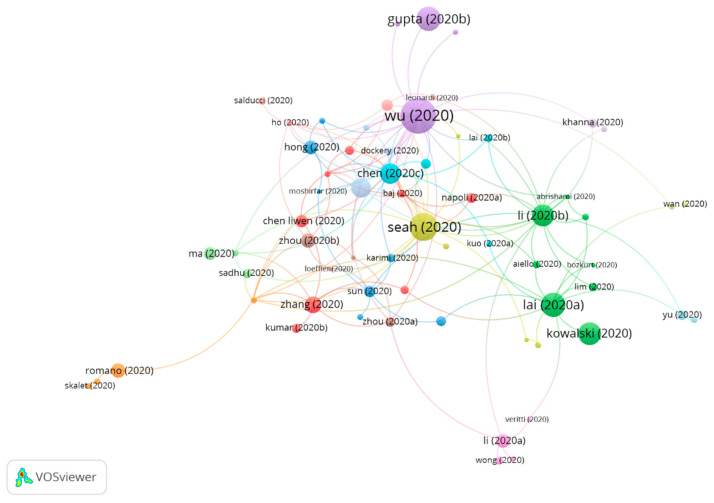
Core Publications on the COVID-19 and vision citation network.

A total of 117 publications were found with four or more citations, and the citation network comprises 574, representing 30.01% (Figure 9). Therefore, there is a clear focus on the research topic, the most common one being the diverse ocular manifestations in patients with COVID-19.

## 4. Discussion

The main databases, such as Web of Science or Scopus, allow the creation of citation networks. However, when conducting a systematic review of all the existing literature on a subject, their usefulness is limited, given that they do not provide a general overview of the connection between citations of a group of publications. Therefore, the CitNetExplorer and CiteSpace software were used to visualize, analyze, and explore citation networks of scientific publications, as these offer more detailed analysis when creating citation networks than databases such as Web of Science or Scopus [21].

The main aim of this study was to analyze the existing literature about the impact of COVID-19 at the ocular level. To this effect, the Web of Science database was used. It presents one of the most extensive databases since its search range starts in 1900. However, it should be noted that Web of Science only accepts journals with an international presence that have passed a rigorous selection process.

Thus, by downloading the existing bibliography at WOS, the CitNetExplorer and CiteSpace software allowed us to collect and analyze all the available literature on the impact of COVID-19 in ocular disease. The connection between fields of study and different research groups was also assessed through citation network analysis. To obtain the results, the *clustering* function was used. This function allows the grouping of the publications according to the relationship among citations. The *drilling down* function was also used to conduct a deeper analysis of the existing bibliography of each group. The *core publications* function shows the main publications, i.e., those with a minimum number of citations (≥4 citations). Therefore, these functions make it possible for a complete analysis and study of the research on the field of study to be conducted. The methodology was based on other citation network studies carried out by our research team [49,50,51].

The first publication on eye symptoms in COVID-19 patients was published by Lu et al. [52] in the *Lancet*, in a letter to the editor where conjunctivitis was found as the first symptom in a pulmonologist, after having been in a hospital in Wuhan. Nevertheless, the first articles were only published in March 2020. One publication that should be noted is the one published by Chen et al. [42] in *Acta Ophthalmologica*, where they found that 27 out of the 535 patients with COVID-19 had conjunctival congestion (four of them as an initial symptom). The average duration of conjunctival congestion was 5.9 to 4.5 days. They also found that some patients with COVID-19 had chronic ocular diseases, such as conjunctivitis (6.2%), xerophthalmia (4.5%), and keratitis (2.6%). Therefore, conjunctival congestion is one of the ocular symptoms related to COVID-19 and could occur as an initial symptom. Frequent hand-to-eye contact may be a risk factor for conjunctival congestion in patients with COVID-19.

Another study was published by Li et al. [32] in the *British Journal of Ophthalmology*, comparing SARS-CoV-2 with other types of coronaviruses. In doing so, it was found that no ocular involvement has been described with either MERS-CoV or SARS-CoV, although PCR-RT on tears from patients with SARS-CoV infection demonstrated the presence of the virus. There is also evidence that some coronavirus can occasionally cause conjunctivitis in humans. Human coronavirus NL 63 (HCoV-NL63) was first detected in an infant with bronchiolitis and conjunctivitis. Subsequently, 17% of 28 cases of children with confirmed HCoV-NL63 infections had conjunctivitis [53,54,55,56,57,58].

One journal with a particularly high number of publications on the impact of COVID-19 at the ocular level is the Indian Journal of Ophthalmology, which occupies the 68th place in the ophthalmology category and has an impact factor of 1.25. This is related to the fact that India is one of the countries with the highest number of publications in this area of research, because of the potential for extensive nationwide transmission based on the current health management system and a population that rivals China [59]. Furthermore, the journal with the highest impact factor is *Ophthalmology*, 8.47. However, it must be noted that the impact factor is a critical index that informs about the importance of journals, but it is not an absolute measurement index. The main difference between both is that the latter is based on the impact of the research results, as well as the physical and intellectual contributions of the authors [60]. At the beginning of the pandemic, the first articles were published in China. However, as it advanced, other countries such as India, Italy, and the United States increased their number of publications. This is related to increased concern among healthcare workers and front-line politicians due to the language barrier. Today, research is mainly focused on symptoms, as well as finding an effective treatment and vaccine against COVID-19. This explains why research is being done in this field in countries with higher incomes and therefore better infrastructures, leading to a growing number of publications. We assume that lower-income countries focus on other research fields, such as transmission or epidemiology, although with fewer publications [26,27].

Today, numerous studies analyze the transmission of SARS-CoV-2 at the ocular level.

This agrees with the results obtained in our study that the most cited publications are those that analyze the most frequent ocular symptoms in patients with COVID-19.

On the one hand, in a study by Sun et al. [48], the authors considered that the risk of transmission of the virus by the ocular surface was unlikely, due to the low prevalence of SARS-CoV-2 on the ocular surface and because of conjunctivitis related with COVID-19. However, they warned that the virus can be transmitted during ophthalmic practice. In the study by Peng et al. [61], the authors stated that the detection of SARS-CoV-2 RNA in the tears and conjunctival secretions of COVID-19 patients with conjunctivitis could be coincidental, instead of indicating SARS-CoV-2 conjunctiva infection as the cause of conjunctivitis. Despite this, there is enough anatomical evidence justifying the possibility that the ocular surface is a route of transmission of SARS-CoV-2 [62]. Thus, in Dawood’s study [63], the existence of ACE2 was shown in conjunctiva, cornea, and limbus after immunohistochemistry analysis. Moreover, conjunctival surgical specimens show ACE2 expression in the conjunctival epithelium. For this reason, ACE2 in the host cell is considered to act as a receptor for SARS-CoV-2. Likewise, cellular protease TMPRSS2 is confirmed to facilitate viral entry after the union of SARS-CoV-2 protein S with ACE2. More specifically, protein S is divided into two subunits, S1 and S2, by an extracellular protease. While S1 binds to ACE2, S2 cleaves further and is activated by TMPRSS2. On the other hand, it has also been related to the fact that the ocular surface, which is comprised of the tear film and the epithelium of the conjunctiva and cornea, is closely linked to the respiratory tract through the nasolacrimal duct. Thus, blinking spreads, mixes, and distributes tears and generates a pumping effect that attracts tears to the lacrimal sac and then into the inferior nasal meatal of the nose [53,64]. Thus, the nasolacrimal duct forms a conduct for viruses to spread between the eye and the upper respiratory tract. However, the direction in which SARS-CoV-2 would spread along in this conduct is unknown. In this regard, Tong et al. [65] examined this connection, using conjunctival-upper respiratory tract irrigation to test for the presence of SARS-CoV nucleotides. Patients self-administered a drop of saline solution into each eye, blinked repeatedly, tilted their head back, and breathed to facilitate drainage into the nasopharynx. These samples were positive in two of the four patients confirmed with SARS-CoV-2.

The overall prevalence of ocular symptoms in patients with COVID-19 is 11.2%, which means that it is not a common finding. However, this prevalence could be an underestimation, since patients with COVID-19 present life-threatening clinical scenarios, which may prevent a detailed ocular examination or relevant background information. Furthermore, in large retrospective studies, ophthalmological examinations were not accurately detailed [6]. The most frequent ocular symptom is conjunctivitis, together with hyperemia, foreign body sensation, chemosis, and epiphora, followed by ocular pain, dry eye, floaters, and palpebral dermatitis [14]. However, other authors found no evidence of significant conjunctival expression of ACE-2 [66]. A recent study showed that no consistent TMPRSS2 existence can be found in conjunctival samples, while it is present in some pterygium samples [41].

A recent report analyzing findings in optical coherence tomography (OCT) in 12 patients who tested positive for COVID-19 showed hyperreflective lesions at the ganglion cell layer and inner plexiform layers. This finding was bilateral and was present in all patients, being more prominent in the papillomacular bundle [67]. It should also be noted that elevated serum levels of complement C3 have also been related to an increased risk of developing diabetic retinopathy, nephropathy, and neuropathy, through endothelial dysfunction and thrombosis [68]. Immunohistochemistry in the human eye has shown that the ciliary body, choroid, retina, and retinal pigment epithelium (RPE) contain significant levels of ACE receptors [69]. Since COVID-19 can target vascular pericytes expressing ACE-2, a viral infection could lead to an endothelial cell dysfunction complement-mediated, microvascular damage and thus the involvement of the ocular circulation. C3a and C5a, small cleavage fragments (approximately 10 KDa) are released through complement activation. That is, they are powerful mediators of inflammation. They are considered anaphylatoxins and act as cell activators presenting nanomolar affinity. The interaction between C3a and C5a with their respective receptors on vascular endothelium is associated with over-expression of ICAM-1 by these vascular endothelial cells. ICAM-1 increases the activation of vascular endothelial cells [70]. COVID-19-associated coagulopathy may predispose to a spectrum of thromboembolic events.

Neurological alterations in patients with COVID-19 include polyneuritis, Guillain–Barré syndrome (GBS), meningitis, encephalomyelitis, and encephalopathy [71]. Oculomotor nerve palsy could be triggered by the direct invasion of the virus, inflammatory factors related to viral infection, or could be secondary to neurological complications such as GBS, acute disseminated encephalomyelitis, or transverse myelitis [72]. SARS-CoV-2 can trigger GBS via the molecular mimicry mechanism due to similarities between amino acid sequences of SARS-CoV2 protein and neuronal membrane gangliosides. One of the prominent assumptions about the underlying pathological mechanisms of GBS is molecular mimicry. This is a process in which antibodies created to fight against viruses or other pathogens bind to gangliosides on the surface membranes of peripheral sensory and motor neurons. This causes immune-mediated damage to the myelin sheath and/or axons. The SARS-CoV-2 infection triggers an adaptive immune response in which interactions between T cells and B cells result in the production of specific antibodies against SARS-CoV-2, but a similarity in sequences or structure of viral peptides and gangliosides (molecular mimicry). This cross-reactivity in SARS-CoV-2 infected individuals, with appropriate HLA-typing due to similarity between amino acid sequences of SARS-CoV2 proteins and neuronal membrane gangliosides, lead to a loss of immunotolerance [73].

Although animal models suggest that eye injuries could include optic neuritis, an increased incidence of cases of ischemic or inflammatory optic neuropathies associated with COVID-19 has not yet been reported in the literature [29].

In turn, the most frequent ocular complications in ICU patients are superficial disorders, ranging from mild conjunctival irritation to severe infectious keratitis. This is because these patients have several risk factors for superficial disorders, some of them related to treatments, while others are related to the ICU environment, for example, exposure to many potentially multiresistant bacteria [74,75].

Chloroquine (CQ) and hydroxychloroquine (HCQ) have been widely adopted in the clinical setting for the treatment of SARS-CoV-2 infection. However, the proposed doses (CQ: 1000 mg/day for 10 days; HCQ: 800 mg on the first day and then 400 mg/day for 5 days) are much higher than the recommended maximum safe daily doses of both agents (CQ: ≤2.3 mg/kg/day; HCQ: ≤5.0 mg/kg/day). Exposure to safe doses can cause retinal toxicity within five years, so it is considered that exposure to high doses for a short period of time could also cause retinal damage [76].

Second-generation antiretroviral drugs (lopanivir and ritnovaris) used for HIV treatment can cause long-term pigmentary changes in the macula, resulting in severe vision loss [77]. However, in most cases of COVID-19, the of treatment lasts between five and seven days, so retinal toxicity is unlikely to occur [78].

Thus, social distancing has led companies to perform operations remotely and it is speculated whether there will be a “new normality” after this pandemic. Hence, that would imply telehealth for medicine. Teleophthalmology has been repeatedly demonstrated to be of help in the detection and treatment of a variety of eye conditions in adults and children. It has the potential to increase access to primary and specialized care and overcome the unique barriers that the COVID-19 pandemic has created. Until now, tele-ophthalmology has been used primarily for screening for diabetic retinopathy, whose prevalence continues to increase, diagnosis of glaucoma, and macular degeneration monitoring. Ophthalmology clinics equipped with remote sensing devices, functional on their own or that could be operated by minimally trained staff, can bridge a gap in the availability of treatment in prolonged periods of quarantine and isolation, along with patients with COVID-19. Therefore, in the future, thanks to the improvements in image processing, as well as better integration with electronic medical records, teleophthalmology will probably become a much more accepted and more widely used modality, particularly in circumstances in which social distancing measures are recommended [79].

## 5. Conclusions

In conclusion, this study offers a comprehensive and objective analysis of the main papers on the impact of COVID-19 at an ocular level. Furthermore, by using the Web of Science database and the Citation Network Explorer software, it was possible to visualize, analyze, and explore the most cited articles and the existing citation networks to date.

In this study, three main groups have been found on the impact of COVID-19 at the ocular level (ocular manifestations, teleophthalmology, and personal protective equipment). Eye symptoms being the most researched topic. In addition, it has been found that the most affected countries are those that present a greater number of publications.

Thus, ocular symptoms are a common finding in COVID-19 patients. These are more frequent in ICU patients due to various risk factors that can alter the ocular surface. That is, in patients with mild symptoms it is rare to find them. In addition, many articles have found the presence of COVID-19 in the tear, so it can be transmitted through this route.

For this reason, together with social distancing, teleophthalmology has been created, showing potential for the treatment and monitoring of patients.

The number of citation network studies has been increasing because it is the only method of analysis that provides a global overview of the different fields of study within a specific topic. Moreover, the CitNetExplorer software facilitates the analysis of all existing studies on a given matter, as it allows for more detailed research. This might change how research is conducted in different fields of study.

## Figures and Tables

**Table 1:** Characteristics of the main countries.

Group	Color	Main Countries	Publications	Centrality	Degree	Half-Life	Connections
1	Red	EE. UU	105	0.01	5	−0.5	60
2	Green	Germany	22	0.04	16	−0.5	43
3	Blue	Switzerland	6	0.12	17	−0.5	14
4	Yellow	The Netherlands	7	0.15	13	−0.5	12

**Table 2:** The 10 research areas with the highest number of publications.

Category	Frequency	Centrality	Degree	HalfLife
Ophthalmology	263	0.18	11	−0.5
General & Internal Medicine	138	0.00	11	−0.5
Research & Experimental Medicine	78	0.25	17	−0.5
Infectious Diseases	53	0.06	10	−0.5
Neurosciences & Neurology	31	0.10	8	−0.5
Public, Environmental & Occupational Health	25	0.03	5	−0.5
Surgery	18	0.03	5	−0.5
Clinical Neurology	8	0.04	6	−0.5
Pediatrics	7	0.00	3	–0.5
Pharmacology & Pharmacy	7	0.07	10	–0.5

**Table 3:** The 10 authors with the highest number of publications.

Author	Number of Publications	*H* Index	Total Citations	Citation Average	Centrality	Degree	Connections
Sharma N	9	2	12	1.56	0.00	9	175
Bandello F	8	4	29	5.17	0.00	8	151
Agrawal R	7	3	167	30.00	0.08	5	428
Shetty R	7	2	12	2.67	0.01	11	74
Sachdev MS	7	2	12	2.00	0.00	8	138
Sinha R	5	2	10	2.40	0.00	8	125
Arriola-Villalobos P	4	2	11	3.50	0.00	1	144
Khamar P	4	2	8	2.67	0.00	8	43
D´souza S	4	1	11	2.50	0.00	9	50
Levy J	4	1	7	3.00	0.00	2	53

**Table 4:** The 10 institutions with the highest number of publications.

Category	Frequency	Centrality	Degree	Half-Life	Connections
All India Institute of Medical Sciences	15	0.02	15	−0.5	74
LV Prasad Eye Institute	13	0.02	13	−0.5	24
Ctr Sight	12	0.04	15	−0.5	46
Harvard Medical School	10	0.10	8	−0.5	15
Tan Tock Seng Hospital	9	0.11	18	−0.5	59
Johns Hopkins University	8	0.03	2	−0.5	6
Stanford University	7	0.39	11	−0.5	11
National University of Singapore	7	0.10	15	−0.5	54
University of Miami	7	0.21	6	−0.5	9
Sankara Nethralaya	6	0.03	15	−0.5	33

**Table 5:** The 10 journals with the highest number of publications.

Journal	Total Publications	Impact Factor (2019)	Quartile Score	SJR (2019)	Citations/Docs (2 years)	Total Citations (2019)	*H* Index	Country
Indian Journal of Ophthalmology	53	1.25	Q4	0.48	1.239	1082	47	India
Graefe’s Archive for Clinical and Experimental Ophthalmology	17	2.39	Q2	1.26	2.811	2273	96	Germany
Ocular Immunology and Inflammation	14	2.11	Q2	0.78	2.331	844	53	United Kingdom
Ophthalmology	14	8.47	Q1	4.42	8.476	6778	229	Netherlands
Current Opinion in Ophthalmology	12	2.98	Q1	1.23	3.288	870	83	United States
Ophthalmologe	11	0.74	Q4	0.26	0.655	306	38	Germany
Eye	11	2.45	Q2	1.15	2.689	1930	93	United Kingdom
Journal francais d´Ophalmologie	11	0.64	Q4	0.27	0.493	287	29	France
Clinical Ophthalmology	10	-	-	0.96	2.077	2033	50	New Zeland
Acta Ophthalmologica	8	3.36	Q1	1.42	3.304	2369	82	United States

**Table 6:** The 30 most used keywords.

Keyword	Frequency	Centrality	Degree	Total Link Strength
COVID-19	168	0.01	6	485
Coronavirus	85	0.03	11	352
SARS-COV-2	74	0.05	9	309
Ophthalmology	45	0.03	6	155
Conjunctivitis	36	0.01	6	154
Telemedicine	24	0.02	12	77
Infection	23	0.06	8	96
Transmission	20	0.02	9	117
SARS	19	0.03	7	105
Pandemic	19	0.00	2	52
Coronavirus disease 2019	18	0.00	3	71
Outbreak	15	0.01	6	83
Eye	15	0.00	1	66
Pneumonia	14	0.03	11	99
ACE 2	14	0.08	17	88
Ocular surface	14	0.05	9	70
Respiratory syndrome coronavirus	12	0.00	4	62
Chloroquine	11	0.00	9	51
Diagnosis	11	0.04	11	74
Hydroxychloroquine	10	0.02	11	55
Teleophthalmology	10	0.07	11	43
Conjunctiva	9	0.01	9	60
Retinopathy	9	0.02	9	35
Receptor	9	0.00	4	56
Lockdown	9	0.00	1	23
Ocular manifestation	9	0.00	3	31
Disease	9	0.00	3	33
Guidelines	9	0.00	1	26
Inhibition	8	0.06	12	29
Retina	8	0.03	7	34

**Table 7:** Characteristics of most-used keywords.

Cluster	Color	Main Keywords	Topic	%
1	Red	Covid-19, telemedicine, pandemic, teleophthalmology, telehealth	Benefits of teleophthalmology in patients with COVID-19	2.39
2	Green	SARS-COV-2, respiratory syndrome coronavirus, transmission, infection, pneumonia	Transmission methods	1.74
3	Blue	Ophthalmology, conjunctivitis, sars, outbreak, virus	Ocular manifestations	1.30
4	Yellow	Coronavirus, coronavirus disease 2019, personal protective equipment, children, disease	Personal protective equipment	0.98
5	Violet	Prevention, inhibition, chloroquine, hydroxychloroquine, retinal toxicity	Ocular side effects of treatment in patients with COVID-19	0.98

**Table 8:** Description of the 20 most cited publications on COVID-19 and vision.

Author	Title	Journal	Year	Citation Index	Links
Wu et al. [14]	Characteristics of Ocular Findings of Patients With Coronavirus Disease 2019 (COVID-19) in Hubei Province, China	JAMA Ophthalmol. 1 May 2020;138(5):575–578	2020	229	94
Seah et al. [29]	Can the Coronavirus Disease 2019 (COVID-19) Affect the Eyes? A Review of Coronaviruses and Ocular Implications in Humans and Animals	Ocul Immunol Inflamm. 2 April 2020;28(3):391–395	2020	143	66
Lai et al. [30]	Stepping up infection control measures in ophthalmology during the novel coronavirus outbreak: an experience from Hong Kong	Graefes Arch Clin Exp Ophthalmol. May 2020;258(5):1049–1055.	2020	111	42
Gupta et al. [31]	Extrapulmonary manifestations of COVID-19	Nat Med. July 2020;26(7):1017–1032	2020	111	2
Li et al. [32]	Novel Coronavirus disease 2019 (COVID-19): The importance of recognising possible early ocular manifestation and using protective eyewear	Br J Ophthalmol. March 2020;104(3):297–298.	2020	90	39
Chen et al. [33]	Ocular manifestations of a hospitalised patient with confirmed 2019 novel coronavirus disease	Br J Ophthalmol. June 2020;104(6):748–751.	2020	79	48
Colavita et al. [34]	SARS-CoV-2 Isolation From Ocular Secretions of a Patient With COVID-19 in Italy With Prolonged Viral RNA Detection	Ann Intern Med. 4 August 2020;173(3):242–243.	2020	71	37
Zhang et al. [35]	The evidence of SARS-CoV-2 infection on ocular surface	Ocul Surf. July 2020;18(3):360–362.	2020	51	31
Zhou et al. [36]	Ocular Findings and Proportion with Conjunctival SARS-COV-2 in COVID-19 Patients	Ophthalmology. July 2020;127(7):982–983.	2020	40	26
Romano et al. [37]	Facing COVID-19 in Ophthalmology Department	Curr Eye Res. June 2020;45(6):653–658	2020	39	19
Tostmann et al. [38]	Strong associations and moderate predictive value of early symptoms for SARS-CoV-2 test positivity among healthcare workers, the Netherlands, March 2020	Euro Surveill. April 2020;25(16):2000508	2020	39	4
Olivia Li et al. [39]	Preparedness among Ophthalmologists: During and Beyond the COVID-19 Pandemic	Ophthalmology. May 2020;127(5):569–572	2020	36	22
Hong et al. [40]	Evaluation of ocular symptoms and tropism of SARS-CoV-2 in patients confirmed with COVID-19	Acta Ophthalmol. 26 April 2020;10.1111/aos.14445.	2020	34	22
Ma et al. [41]	Expression of SARS-CoV-2 receptor ACE2 and TMPRSS2 in human primary conjunctival and pterygium cell lines and in mouse cornea	Eye (Lond). July 2020;34(7):1212–1219	2020	31	15
Chen et al. [42]	Ocular manifestations and clinical characteristics of 535 cases of COVID-19 in Wuhan, China: a cross-sectional study	Acta Ophthalmo. December 2020;98(8):e951–e959.	2020	27	18
Scalinci et al. [43]	Conjunctivitis can be the only presenting sign and symptom of COVID-19	DCases. 2020;20:e00774.	2020	26	20
Saleem et al. [44]	Virtual Ophthalmology: Telemedicine in a COVID-19 Era	Am J Ophthalmol. August 2020;216:237–242	2020	23	7
Korobelnik et al. [45]	Guidance for anti-VEGF intravitreal injections during the COVID-19 pandemic	Graefes Arch Clin Exp Ophthalmol. June 2020;258(6):1149–1156.	2020	22	7
Mishra et al. [46]	The impact of COVID-19 related lockdown on ophthalmology training programs in India—Outcomes of a survey	Indian J Ophthalmol. June 2020;68(6):999–1004.	2020	21	10
Zhou et al. [47]	ACE2 and TMPRSS2 are expressed on the human ocular surface, suggesting susceptibility to SARS-CoV-2 infection	Ocul Surf. October 2020;18(4):537–544	2020	19	15

**Table 9:** Information on the citation networks of the 3 main groups.

Main Cluster	Number of Publications	Number of Citation Links	Number of Citations Median (Range)	Number of Publications with ≥4 Citations	Number of Publications in the 100 Most Cited Publications
Group 1	158	604	0 (0–94)	37	53
Group 2	68	84	1 (0–7)	10	32
Group 3	60	87	0 (0–40)	9	15

**Table 10:** Main citation network groups from the subcluster in group 1.

Sub-Cluster	1	2	3
Nº of publications	103	27	20
Nº of citation links	381	30	17
Most cited publication	Wu et al. [14]	Sun et al. [48]	Romano et al. [37]
Main Keywords	Conjunctivitis, retina, glaucoma	Sars-cov-2, Hydroxychloroquine, retinal toxicity	Ocular manifestation, neuro-ophthalmology, optic neuritis
Topic of discussion	Ocular characteristics in COVID-19 patients	Ocular risks following the administration of ritonavir, chloroquine and hydroxychloroquine	Neuroophthalmic manifestations in COVID-19 patients
Conclusion	A wide spectrum of ocular manifestations might occur, ranging from anterior segment pathologies such as conjunctivitis and anterior uveitis to affectations compromising vision, such as retinitis and optical neuritis.	Evidence to date shows that extreme dosage accelerates retinal toxicity, but within a period of time which is likely to be of many months rather than a few days.	Neuroophthalmological signs and symptoms may appear isolated or associated to neurological disorders. Most common manifestations include headaches, eye pain, vision impairment, diplopia, and secondary cranial pairs palsy associated to Miller Fisher syndrome, Guillain–Barré syndrome or encefalitis and nystagmus.
